# Diffuse Gastrointestinal Polyposis Revealing Mantle Cell Lymphoma: A Case Highlighting a Diagnostic Pitfall

**DOI:** 10.7759/cureus.105331

**Published:** 2026-03-16

**Authors:** Rached Radwan, Hayab Karaki, Bouchra Hamade, Abbas Rachid, Abbas Bahr

**Affiliations:** 1 Gastroenterology and Hepatology, Lebanese University, Beirut, LBN; 2 Faculty of Medical Sciences, Lebanese University, Beirut, LBN; 3 Internal Medicine, Lebanese University, Beirut, LBN; 4 Gastroenterology and Hepatology, Bahman University Hospital, Beirut, LBN

**Keywords:** gastrointestinal non-hodgkin lymphoma, gastrointestinal polyposis, mantle cell lymphoma, multiple lymphomatous polyposis, non hodgkin's lymphoma

## Abstract

Mantle cell lymphoma (MCL) is an uncommon subtype of B-cell non-Hodgkin lymphoma characterized by heterogeneous clinical behavior and frequent extranodal involvement. Gastrointestinal (GI) manifestations are well recognized but often underdiagnosed, particularly when they present as multiple lymphomatous polyposis (MLP), which can closely resemble benign or hereditary polyposis syndromes. We report the case of a 47-year-old man presenting with chronic abdominal pain, diarrhea, pruritus, and unintentional weight loss. Endoscopic evaluation revealed diffuse sessile polypoid lesions throughout the GI tract, initially raising concern for familial adenomatous polyposis. Histopathological examination with immunophenotyping demonstrated MCL. Staging studies confirmed isolated GI involvement without distant organ infiltration. The patient was treated with rituximab-based combination immunochemotherapy, resulting in significant clinical improvement and partial endoscopic regression of lesions after two cycles. This case highlights GI-predominant MCL as a critical diagnostic consideration in adults presenting with diffuse polyposis. Histologic confirmation with immunohistochemistry is essential to avoid misdiagnosis and delays in systemic therapy. Early recognition of this rare presentation enables prompt treatment and improved outcomes.

## Introduction

Mantle cell lymphoma (MCL) is a rare and biologically distinct subtype of B-cell non-Hodgkin lymphoma (NHL), accounting for approximately 5-10% of all NHL cases, predominantly affecting middle-aged to older adults with a marked male predominance [1,2]. Although MCL typically follows a rapidly progressive clinical course, indolent variants have been described. The disease most often presents at an advanced stage, characterized by generalized lymphadenopathy and frequent extranodal involvement, including the bone marrow, peripheral blood, spleen, and gastrointestinal (GI) tract [1,2].

At the molecular level, MCL is defined by the reciprocal chromosomal translocation t(11;14) (q13;q32), which juxtaposes the immunoglobulin heavy chain (IGH) locus with CCND1, resulting in cyclin D1 overexpression and cell cycle dysregulation. This pathognomonic translocation is detectable in the vast majority of cases and constitutes a central feature for diagnostic confirmation [1,2].

Histopathologically, MCL is composed of monomorphic small- to medium-sized lymphocytes that consistently express CD20, CD5, and cyclin D1, while lacking CD23 and CD10, distinguishing it from other small B-cell lymphomas [3]. Prognostic evaluation incorporates markers such as the Ki-67 proliferation index and molecular aberrations, including TP53 mutations, which are associated with increased biological aggressiveness and poorer clinical outcomes [4].

Extranodal involvement of the GI tract is a well-recognized manifestation of MCL. Endoscopically, the most characteristic presentation is multiple lymphomatous polyposis (MLP), defined by numerous polypoid lesions involving contiguous or discontinuous segments of the GI mucosa. First described in the context of MCL, MLP is now regarded as the intestinal counterpart of nodal disease. Lesions may involve any portion of the GI tract and can closely mimic other polyposis syndromes or inflammatory conditions, posing a significant diagnostic challenge [3,5].

Large clinical series indicate that GI involvement may be underrecognized without targeted endoscopic evaluation, as many lesions are not detected on routine imaging modalities such as CT or PET. In patients with confirmed GI MCL, MLP is the predominant endoscopic pattern, and GI involvement correlates with advanced disease stage and higher prognostic indices [5].

Given the overlap with benign or hereditary polyposis syndromes, histopathologic confirmation with immunophenotyping is essential for establishing the correct diagnosis and guiding timely systemic therapy. The present case exemplifies an uncommon GI presentation of MCL that clinically and endoscopically resembled polyposis syndromes, highlighting the critical role of tissue diagnosis and detailed morphologic and immunophenotypic evaluation in management.

## Case presentation

A 47-year-old man, with a 20-pack-year smoking history and a non-alcohol user, with no significant past medical history, presented with a one-year history of chronic burning abdominal pain associated with intermittent nausea and two to three episodes of loose stools per day. During this period, he self-medicated intermittently with rifaximin, with minimal and transient symptomatic relief. He denied fever, chills, night sweats, or diaphoresis. The patient reported progressive postprandial discomfort, reduced oral intake, and unintentional weight loss. He also described generalized pruritus without an associated skin rash. There was no personal or family history of GI or hematologic malignancy.

On initial evaluation, physical examination was unremarkable, with no abdominal tenderness, organomegaly, or peripheral lymphadenopathy. Laboratory investigations demonstrated normal leukocyte counts, hemoglobin within the normal range, and mild thrombocytopenia. Inflammatory markers were modestly elevated, with an erythrocyte sedimentation rate of 28 mm/hr. Liver function tests, including aspartate aminotransferase and alanine aminotransferase, were within normal limits, and serum uric acid was slightly elevated. Abdominal ultrasonography did not reveal any abnormalities of the liver, biliary tract, or pancreas (Table 1).

**Table 1:** Laboratory investigations of the patient during hospitalization WBC: White Blood Cells; INR: International Normalized Ratio; SGPT: Serum Glutamic-Pyruvic Transaminase; GGT: Gamma-Glutamyl Transferase; ESR: Erythrocyte Sedimentation Rate; TG: Triglycerides; LDH: Lactate Dehydrogenase

Parameter	Patient value	Reference range
WBC	8,000	4,500-11,000/mm^3^
Hemoglobin	14.2 g/dL	13.5-17.5 g/d
Platelets	160x10^9^/L	150-400x10^9^/L
Creatinine	0.9 mg/dL	<1 mg/dL
Sodium	137 mEq/L	135-145 mEq/L
Potassium	3.8 mEq/L	3.5-5.2 mEq/L
INR	1.1	0.9-1.3
Lipase	17 U/L	10-140 U/L
SGPT	15 U/L	7-56 U/L
GGT	13 U/L	5-50 U/L
Alk-Ph	35 U/L	30-130 U/L
Total Bilirubine	0.9 mg/dL	0.1-1.2 mg/dL
HCO3	23 mEq/L	22-26 mEq/L
ESR	28 mm/h	2-10 mm/h
Uric acid	7.47 mg/dL	4-8.5 mg/dL
Cholesterol	216 mg/dL	< 200 mg/dL
TG	244 mg/dL	< 150 mg/dL
LDH	210 U/L	140-280 U/L

**Figure 1:**
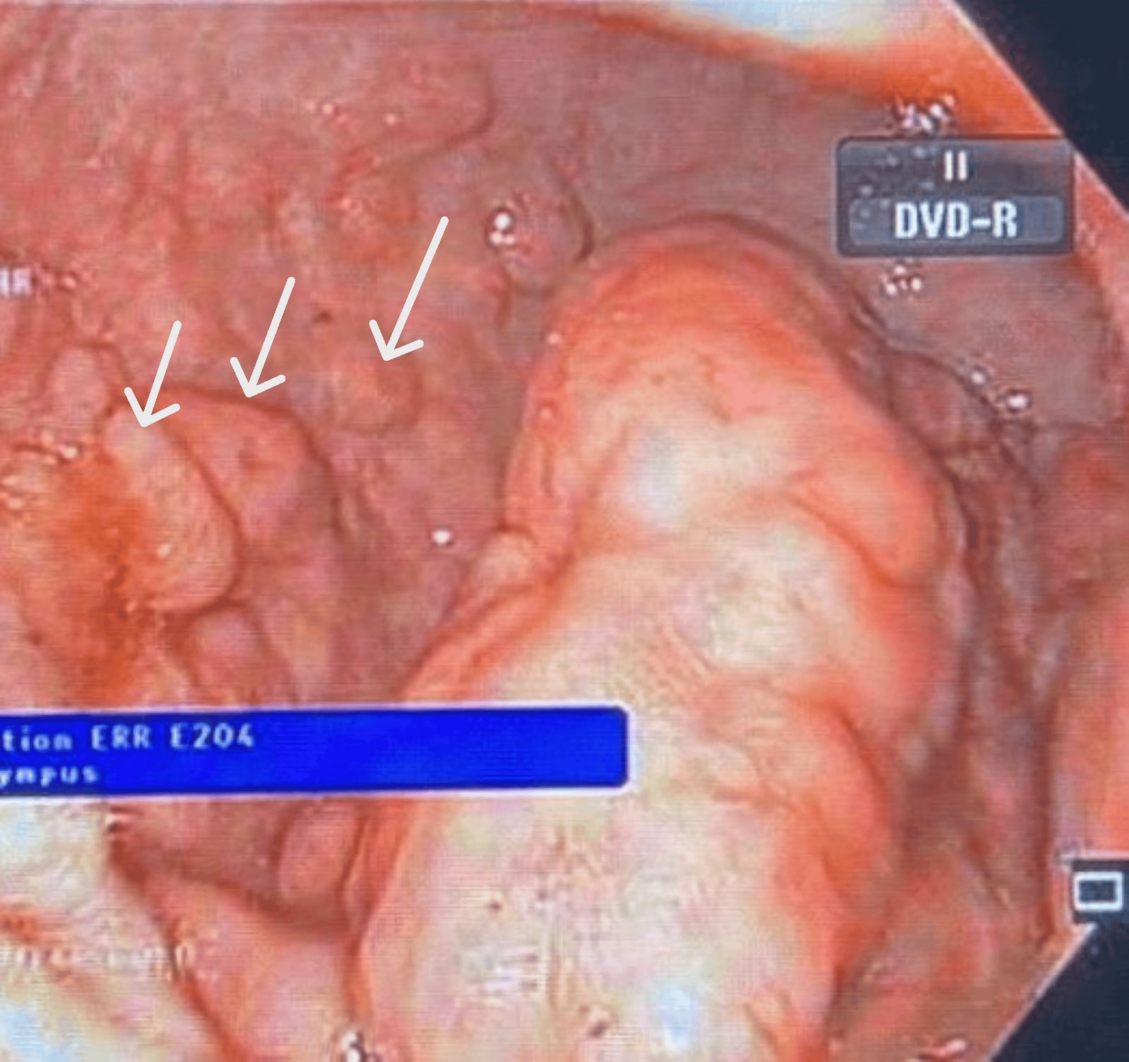
Endoscopic View of Polypoid Gastric Lesions (White Arrows)

**Figure 2:**
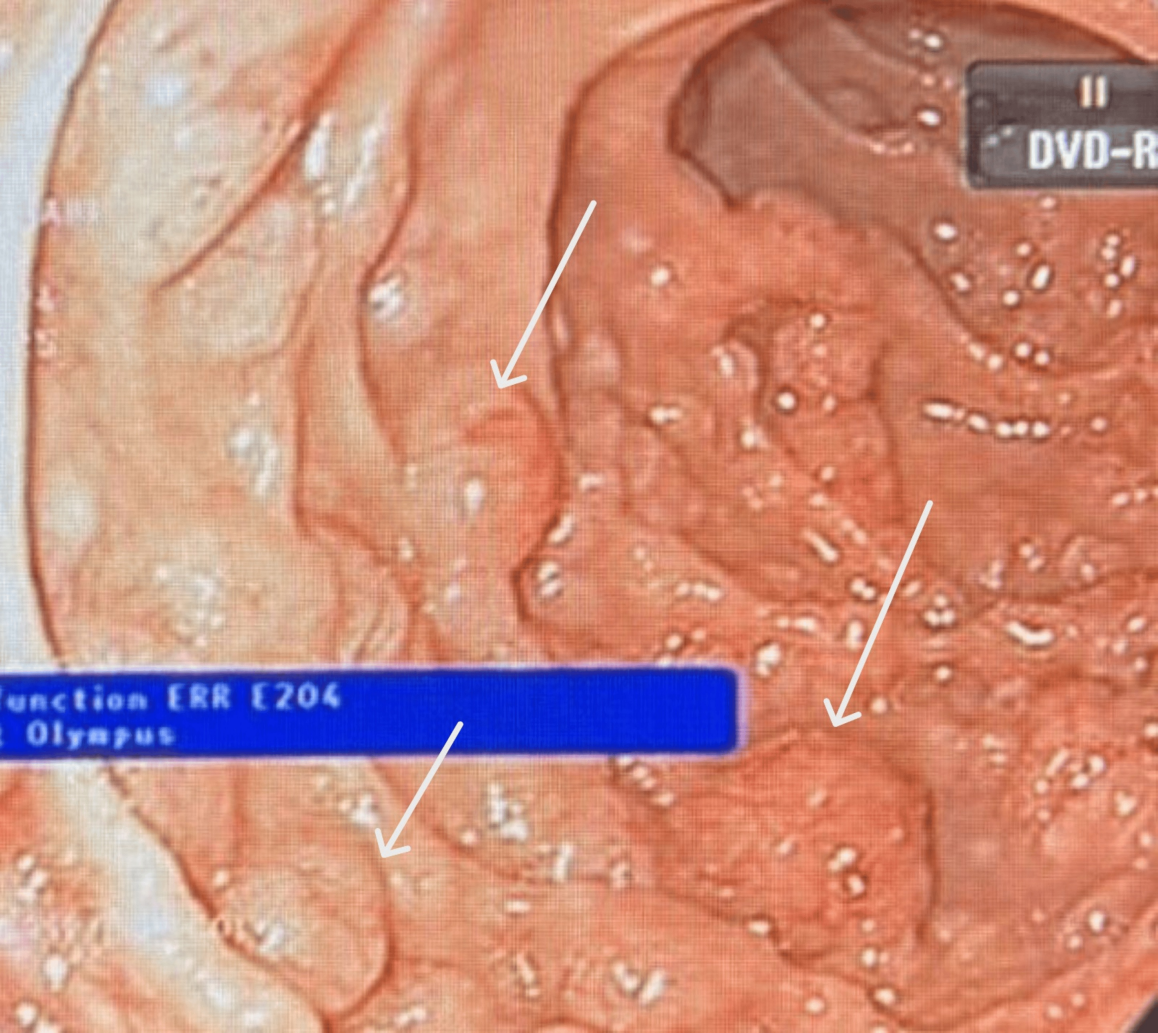
Endoscopic Image Demonstrating Polypoid Lesions in the Duodenum (White Arrows)

**Figure 3:**
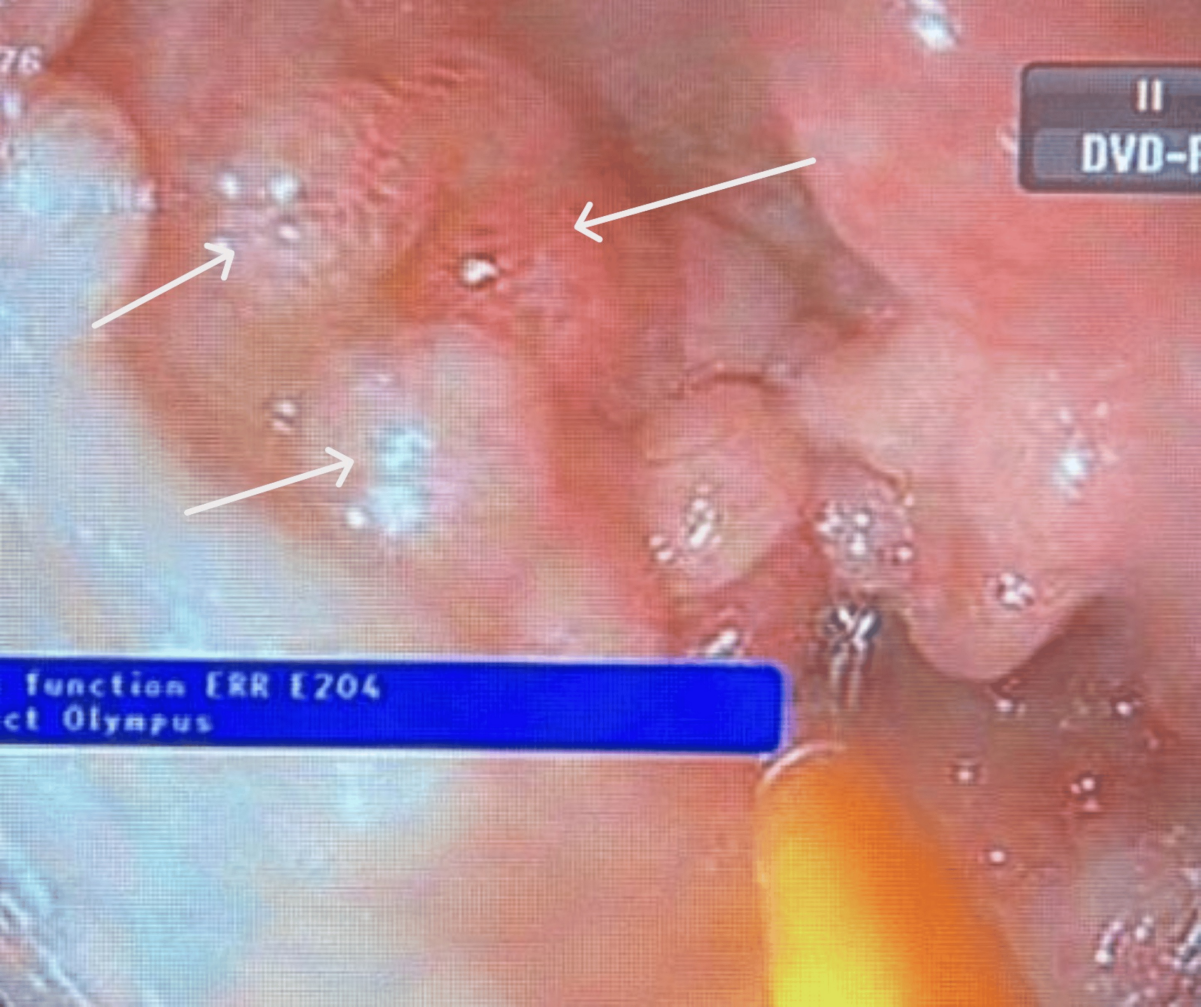
Endoscopic Image Demonstrating Polypoid Lesions in the Ileum (White Arrows)

Given the persistence of GI symptoms and weight loss, upper and lower GI endoscopic evaluation was performed. Esophagogastroduodenoscopy and colonoscopy revealed numerous sessile polypoid lesions diffusely involving the GI tract, raising initial concern for a polyposis syndrome, particularly familial adenomatous polyposis (Figures 1, 2, 3).

**Figure 4:**
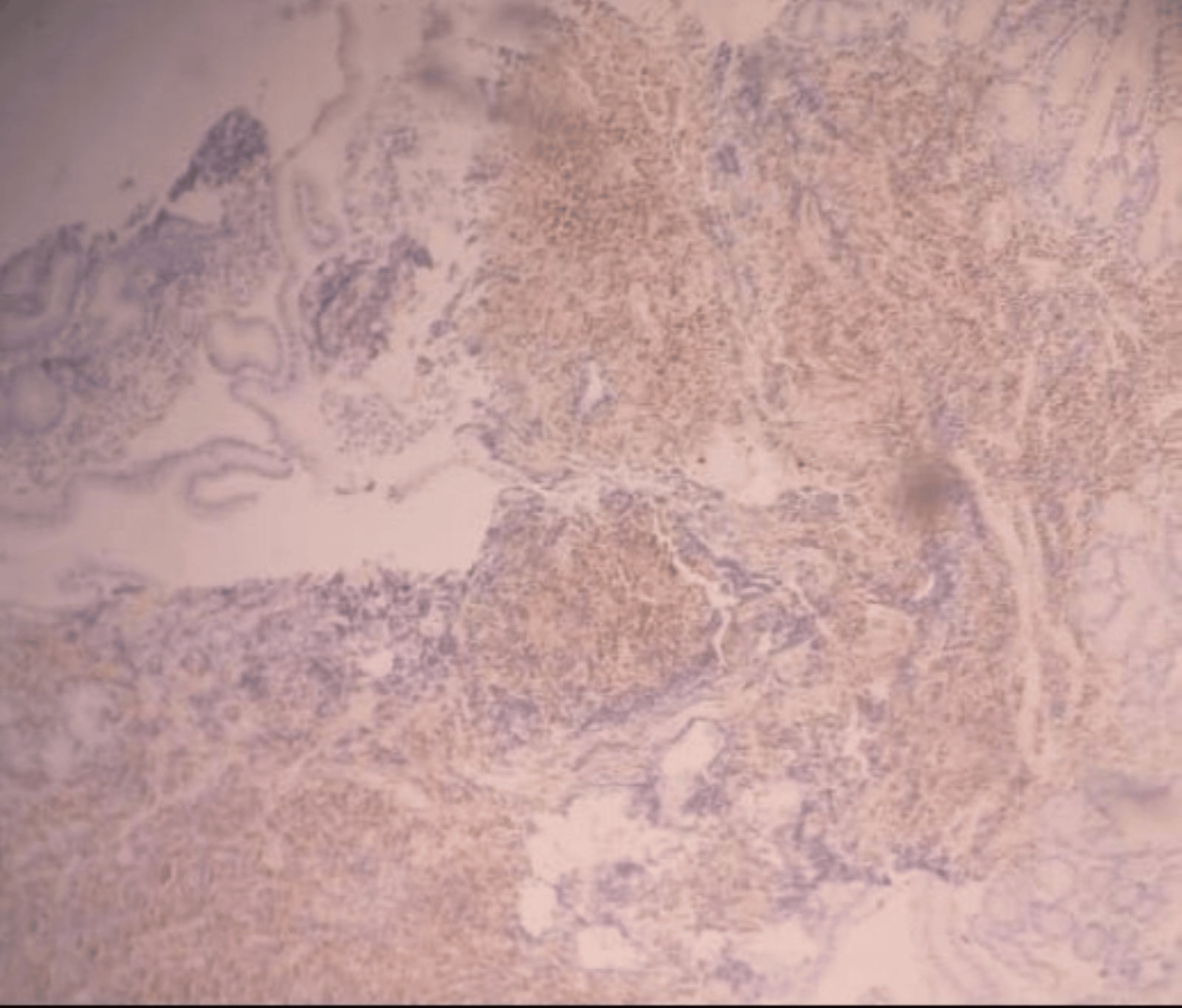
This Figure Demonstrates Immunohistochemical Positivity for Cyclin D1 (CD1), Supporting the Diagnosis of Mantle Cell Lymphoma.

**Figure 5:**
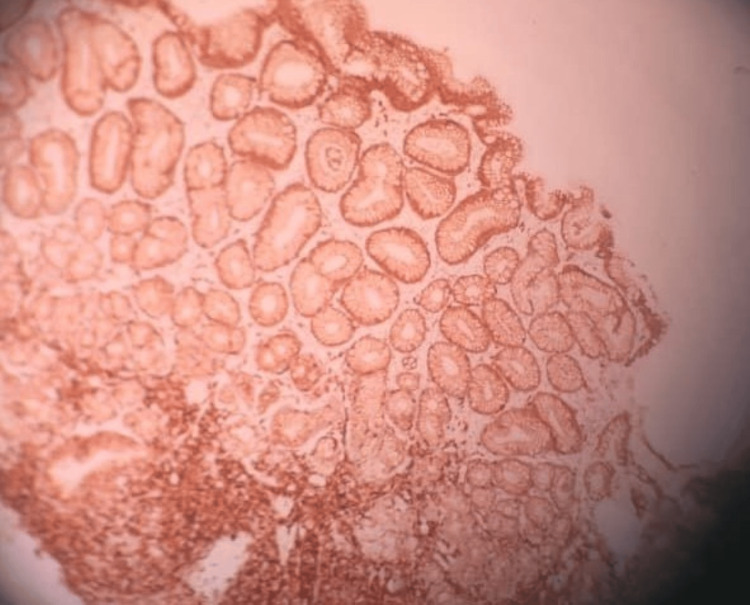
This Figure Demonstrates Immunohistochemical Positivity for Cyclin D5 (CD5), Supporting the Diagnosis of Mantle Cell Lymphoma.

**Figure 6:**
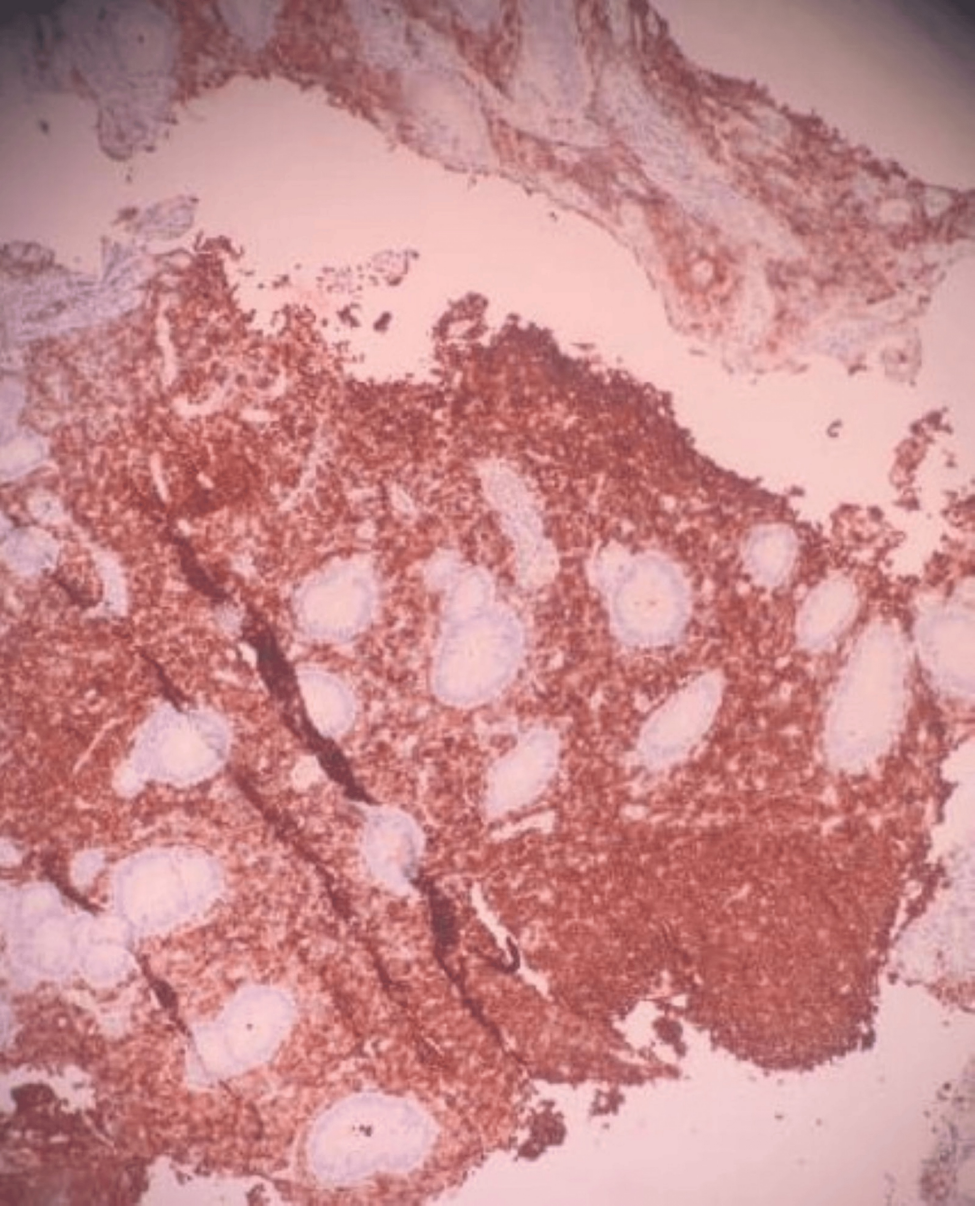
This Figure Demonstrates Immunohistochemical Positivity for Cyclin D79a (CD79a), Supporting the Diagnosis of Mantle Cell Lymphoma.

However, histopathological examination of multiple biopsied lesions from the duodenum, ileum, and colon demonstrated dense lymphoid infiltration consistent with MCL, with immunohistochemical staining positive for Cyclin D1 (CD1), CD79a, and CD5, supporting the diagnosis (Figures 4, 5, 6).

**Figure 7:**
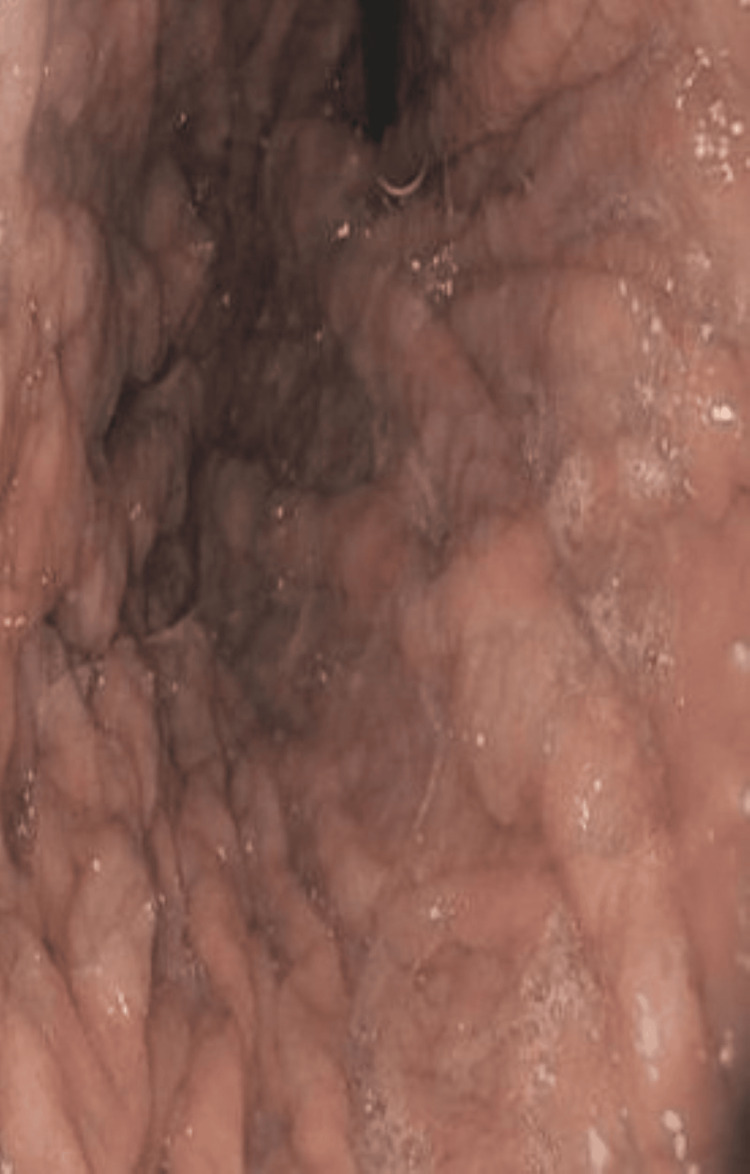
Post-Chemotherapy Endoscopic Resolution of Previously Identified Gastric Polypoid Lesions

The patient was started on combination immunochemotherapy consisting of doxorubicin, vincristine, cyclophosphamide (Endoxan), and rituximab. After two treatment cycles administered on July 11 and August 1, repeat endoscopic assessment demonstrated an approximately 30% reduction in both the size and number of GI polypoid lesions (Figure 7). Clinically, the patient experienced marked symptomatic improvement, with resolution of abdominal pain, nausea, and diarrhea, along with improved oral intake and overall functional status.

## Discussion

MCL demonstrates marked clinical and biological heterogeneity, with GI-predominant disease representing a rare but well-described manifestation [1,2]. The classic GI presentation, MLP, consists of numerous polypoid lesions that may involve any segment of the GI tract, either continuously or discontinuously [5]. Because these lesions closely resemble hereditary polyposis syndromes or benign inflammatory conditions, misdiagnosis is common, particularly in younger patients without systemic lymphoma features.

In the present case, diffuse sessile polyps initially suggested a benign polyposis syndrome, underscoring the diagnostic challenge of GI-predominant MCL. Importantly, extensive GI involvement may occur even in the absence of significant lymphadenopathy or bone marrow infiltration, and a lack of systemic findings does not exclude lymphoma [5,6]. This highlights the need for a high index of suspicion when evaluating adult-onset polyposis.

Definitive diagnosis relies on histopathologic evaluation with immunohistochemistry and, when available, ancillary studies such as flow cytometry or fluorescence in situ hybridization. The characteristic immunophenotype, CD20-positive, CD5-positive, cyclin D1-positive, and CD10/CD23-negative, allows reliable differentiation of MCL from other small B-cell lymphomas [7,8]. In this patient, histologic confirmation enabled prompt initiation of appropriate systemic therapy.

From a therapeutic perspective, GI involvement does not alter the principle that MCL is a systemic disease requiring systemic treatment. Rituximab-based chemo-immunotherapy remains the standard first-line approach, with regimen selection guided by patient age, comorbidities, and disease risk [1,2]. Newer targeted therapies, including Bruton tyrosine kinase inhibitors, have further expanded treatment options in relapsed or high-risk disease. The favorable clinical and endoscopic response observed in this patient underscores the importance of early diagnosis and timely therapy.

## Conclusions

This case illustrates an uncommon presentation of MCL manifesting as extensive GI polyposis. Adult-onset polyposis is often presumed to be benign and hereditary; however, as demonstrated here, it may represent an underlying lymphoid malignancy. Accurate diagnosis requires careful histopathologic evaluation and immunophenotyping, even in the absence of overt systemic disease. Early recognition of such atypical presentations enables timely initiation of systemic therapy, which can improve clinical outcomes and mitigate complications associated with delayed diagnosis.

Furthermore, this case expands the recognized clinical spectrum of MCL, demonstrating that the disease can present predominantly in extranodal sites, particularly the GI tract. Clinicians should maintain a high index of suspicion for lymphoma in adults presenting with unexplained GI polyposis, ensuring prompt diagnosis and appropriate systemic management.
